# High-frame rate vector flow imaging of the carotid bifurcation

**DOI:** 10.1007/s13244-017-0554-5

**Published:** 2017-05-12

**Authors:** Alfredo Goddi, Chandra Bortolotto, Ilaria Fiorina, Maria Vittoria Raciti, Marianna Fanizza, Elena Turpini, Giulia Boffelli, Fabrizio Calliada

**Affiliations:** 1 Centro Medico SME-Diagnostica per Immagini, 31, Via L. Pirandello, 21100 Varese, VA Italy; 2 Radiology Department, Fondazione IRCCS Policlinico San Matteo Pavia, Pavia, Italy

**Keywords:** Ultrasound, Doppler, Vector flow imaging, Plane wave imaging, Carotid arteries

## Abstract

**Abstract:**

Carotid artery atherosclerotic disease is still a significant cause of cerebrovascular morbidity and mortality. A new angle-independent technique, measuring and visualizing blood flow velocities in all directions, called vector flow imaging (VFI) is becoming available from several vendors. VFI can provide more intuitive and quantitative imaging of vortex formation, which is not clearly distinguishable in the color Doppler image. VFI, as quantitative method assessing disturbed flow patterns of the carotid bifurcation, has the potential to allow better understanding of the diagnostic value of complex flow and to enhance risk stratification. This pictorial review article will show which new information VFI adds for the knowledge of hemodynamics in comparison to the conventional ultrasound techniques.

***Teaching points*:**

• *VFI is an angle-independent technique measuring flow velocities in all directions.*

• *This kind of VFI is based on a plane wave multidirectional excitation technique.*

• *VFI allows quantitative assessment of carotid streamlines progression and visualizes vorticity.*

• *VFI does not allow a precise comprehension of streamlines’ 3D shape.*

• *VFI allows a better understanding of carotid artery complex flows.*

**Electronic supplementary material:**

The online version of this article (doi:10.1007/s13244-017-0554-5) contains supplementary material, which is available to authorized users.

## Introduction

Carotid artery atherosclerotic disease is still a significant cause of cerebrovascular morbidity and mortality [1].

Blood flow alteration and inflammation, in addition to systemic risk factors, are considered possible causes for the development of atherosclerotic lesions [2]. Some studies, performed to analyze the flow in the carotid arteries, showed that the development of arterial plaques is more frequent in the presence of a vortex flow [3, 4]. Color Doppler (CD), for evaluation of flow patterns, and spectral Doppler analysis (PW), for measurement of blood velocities, have been used to detect flow disturbances in the carotid bifurcation [5–9]. Although these studies showed that complex flow patterns are detectable, CD and PW are angle-dependent and only estimate the axial component of blood flow velocity; consequently, the quantification of complex flow is not achievable with conventional ultrasound (US) systems. Moreover, CD is also affected by a limited frame rate, allowing low temporal resolution; PW displays the complete spectrum of velocities through the cardiac cycle, but related to a small sample volume and along a single line only. These limitations explain why, in the last decades, the flow complexity analysis was not used for clinical diagnosis or for long-term prognosis and the Doppler evaluation of abnormal flow velocity has been restricted to the grading of vessel stenosis only [10].

In recent years, some manufacturers tried to introduce different techniques with the aim to better describe the flow complexity in the carotid artery and other vessels. A new angle-independent technique, measuring and visualizing blood flow velocities in all directions, called vector flow imaging (VFI), has been proposed [11]. VFI is an operator-independent technique that can provide more intuitive and quantitative imaging of vortex formation, which is not clearly distinguishable in the CD images. Various VFI methods of estimation principles can be used [12–23]. Among the various methods of estimation suggested, the one based on phase shift estimation with transverse oscillations (TO) [11] and the other based on plane-wave imaging (PWI) [19–23] were implemented on commercial systems, thus ready for clinical application. Almost all vendors are rapidly equipping their US scanners with VFI.

The PWI methods estimate the 2D vector velocity of the flow at higher frame rate than the TO method, allowing better depiction of the complex flows. This educational material was collected by using a system equipped with VFI based on a multi-angle transmission plane waves method, which allows a very high frame rate of about 500 Hz [23]. Such a high frame rate offers a detailed visualization of complex flow by showing even transient events, otherwise undetectable.

This pictorial review will consider which new information VFI adds for the knowledge of hemodynamics in comparison to the conventional US techniques.

### High-frame rate VFI

The high-frame rate VFI is based on PWI. Acquisition of flow vector information at high frame rates is obtained by performing multi-directional transmissions of plane waves; after a single plane wave transmission, multiple image receiving lines are obtained [23]. It allows calculation of the true velocity vectors at any location in a vessel. The dynamic flow is obtained by continuously updating the target’s position according to the calculated velocity. The interleaved transmissions ensure both a highly sensitive vector flow image and a high-resolution B-mode images.

The flow is analyzed by the system for 1.5 s at a pulse repetition frequency (PRF) of 10–15 kHz and at a very high frame rate of 400–600 Hz, depending on the used PRF, allowing to study at least one cardiac cycle. The data are reprocessed automatically by the US system in a 35–36-s clip, generating a sequence of about 600–900 images that can be displayed at a frame rate of 20–30 Hz. The acquired data can be further evaluated in the saved video. Such a high frame rate allows a detailed analysis of hemodynamics. V Flow detects the speed and direction of all blood cells flowing through every point of the region of interest (ROI). There are low-speed cells, high-speed blood cells, and reverse cells flowing through a point in a short moment. It means that the speed measured and displayed by V Flow in a point is the average speed of all blood cells in a precise short moment. Spatiotemporal characteristics of flow can be evaluated visually and quantitatively to asses the specific flow pattern. VFI shows velocity vectors, streamline distribution and vorticity distribution. The streamline distribution uses arrows to indicate the flow direction. The color and length of the arrows show the flow velocity, magnitude and direction (green means low velocities, yellow and orange medium velocities and red higher velocities; the longer the arrows the faster the flow). For quantitative evaluation, velocity curves are available: the maximum velocity vector point curve, automatically detected by the system, and the user-defined vector point curve. Both are displayed at the bottom of the image and show the fluctuating velocities of the flow varying in subsequent cardiac cycles.

### Hemodynamic: Brief review

As demonstrated by basic laws of physics, the flow movement depends on various factors, such as: the pushing force, the pressure gradients and the frictional effect of the viscosity relative to the vessel boundaries and between streamlines sliding at different velocities over each other.

#### Laminar flow

Laminar flow is widespread in the body circulation and is found in fairly large and straight arteries. In presence of laminar flow, the front wave is close to a parabolic profile and the directions of streamlines remain almost parallel to the boundaries.

#### Disturbed flow

In particular conditions, the laminar flow tends to become unstable, mixing with eddies and counter eddies: the initial phase of this transition stage consists of perturbations within the boundary layer interacting with shape discontinuities, in particular, changes of vessel lumen diameter, surface curvatures and roughness or velocity changes and relatively high flow velocity [24, 25]. As a consequence of the velocity difference between the flow and the wall boundary, the boundary layer adjacent to the tissue, also known as shear layer, develops vorticity. In other words, where streamlines separate from the wall, the fluid tends to curl into a vortex. This vortex formation process occurs in vessels presenting significant flow decelerations, like in the carotid sinus [25].

Various disturbed flow features were described: a) helical flow, which is a rotation around an axis of flow; b) recirculation, that consists in movement of streamlines from a forward stream back into a separation zone, for example, beyond a plaque; c) turbulence, meaning a regime characterized by randomly and rapidly fluctuating velocities, which may happen after a straight stenosis, creating unsteady vortices of many different sizes that increase friction and energy dissipation [26]. In certain circumstances, these unusual hemodynamic conditions generate an abnormal biological response. Velocity profile skewing can, in fact, create pockets in which the direction of the wall shear stress oscillates, resulting in the development of atherosclerosis [27].

### Blood flow visualization

Diagnostic US applied at the carotid bifurcation offers the possibility of analyzing both anatomy and hemodynamics.

B-mode US is considered the best method for demonstrating arterial wall thickness and plaques. In severe disease, cross-sectional images of the plaques are difficult to generate because of calcium shadowing or reverberations [10].

**Fig. 1:**
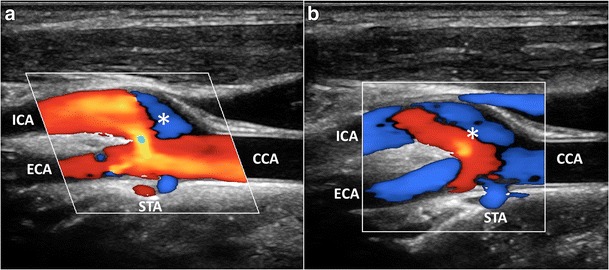
CD of the carotid bifurcation (CB). Complex flow is visible as a mosaic of colors (*), a mixture of velocities and directions; color assignment depends on the US beam direction. (**a**) Color-box steering of 20° to the left. (**b**) Color-box at 90°. CCA = Common carotid artery. ICA = Internal carotid artery. ECA = External carotid artery. STA = Superior thyroid artery

**Fig. 2:**
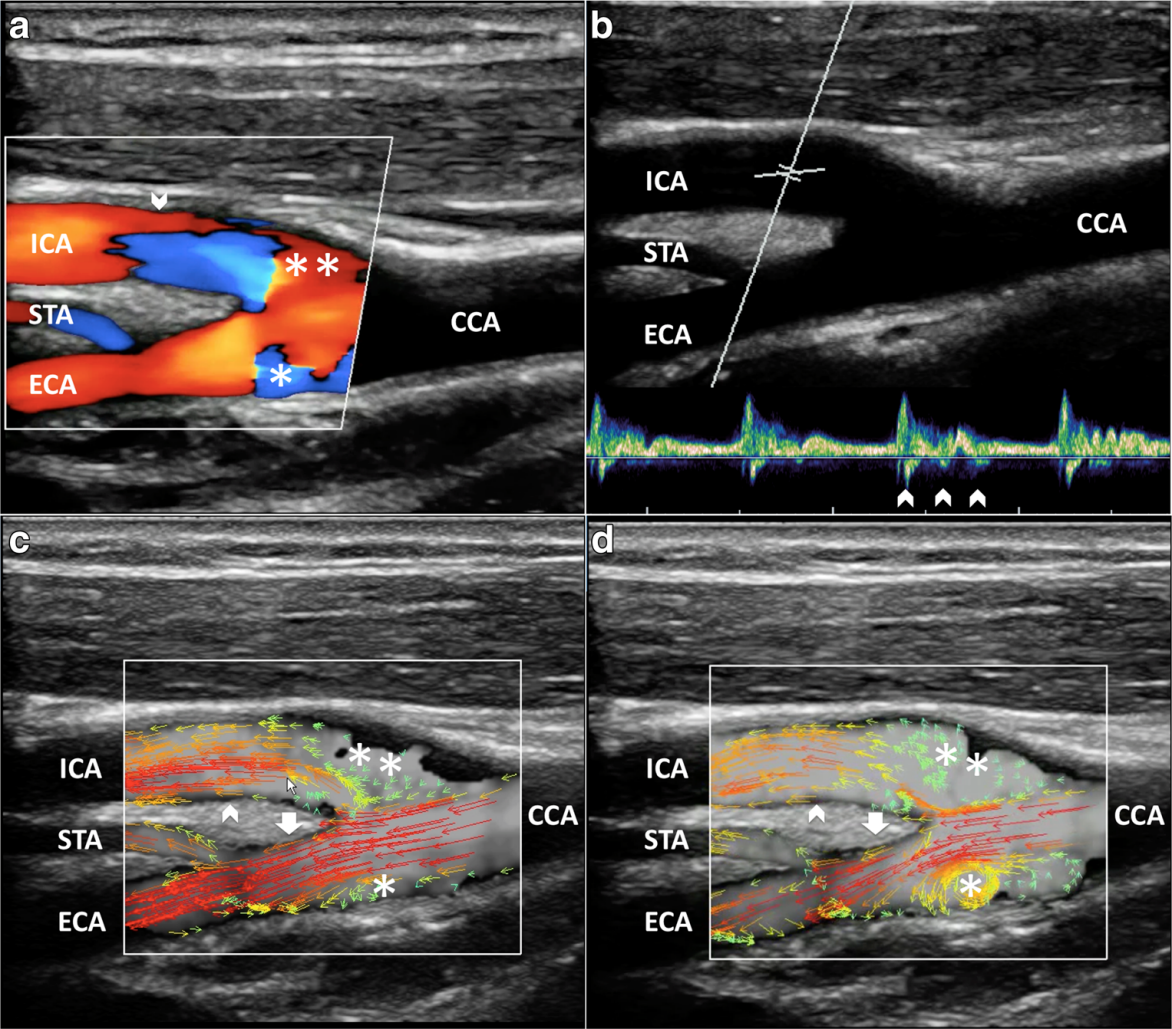
Multimodality Doppler evaluation of the CB. **a**) CD shows reverse flow in the sinus (**), at the ECA entrance (*); in the ICA complex flow (*arrowhead*), the behavior of which is not understandable due to angle dependence. **b**) PW at the ICA: bidirectional flow in the descending systole (*arrowheads*). **c**) VFI at the systolic peak. High-velocity red vectors near the flow divider in ICA (*arrowhead*) and ECA (*white arrow*). **d**) VFI in the descending systole. Multidirectional flow and vortexes (*short green* or *yellow/orange vectors*) in the ICA sinus (**) and at the ECA (*). CCA = Common carotid artery. ICA = Internal carotid artery. ECA = External carotid artery. STA = Superior thyroid artery

CD has been used as a qualitative method for demonstrating abnormal flow patterns where a mosaic of colors indicates a mixture of directions and velocities [8, 9]. CD measures only axial velocities along the line of each ultrasonic beam, displays an ambiguous signal at 90° and suffers from vessel geometry, that may create some misinterpretation of the complex flow pattern: in fact, color assignment depends on the direction from which the vessel is imaged (Figs. 1 and 2a, Vid 2a). PW has been applied to display the normal and abnormal “signature” waveforms that are specific to each vessel. The spectrum shows how the flow velocity and direction of blood cells, present in the Doppler sample volume, vary with time. The peak systolic (PSV) and end-diastolic (EDV) velocities are used to grade carotid stenosis, as arterial narrowing leads to locally increased velocities. Another criterion that could be taken into consideration is the presence of spectral broadening and bidirectional flow, which suggests the existence of flow disturbances [28]. Despite the Doppler spectrum containing the frequency shifts, or velocities, of all the reflectors inside in the region of interest, the small size of the sample volume limits a full overview of the flow behavior and the real streamlines’ movements cannot be distinctly depicted (Fig. 2b). Moreover, the accuracy of Doppler computations depends on precise knowledge of the angle between the US beam and the flow direction. The angle is difficult to estimate with disturbed flow, where streamlines differ from the vessel course. In such condition, post-stenotic disturbed flow can lead to overestimation of velocities, because of incorrect estimation of the angle, even using CD as a guide [10]. Unlike other techniques, VFI measures the axial and transverse vector velocity components and direction of the blood through the vessel, providing both spatial and temporal vector information over the entire carotid bifurcation without the need of any angle correction [11] (Fig. 2c, Vid 2c).

#### VFI laminar flow pattern

**Fig. 3:**
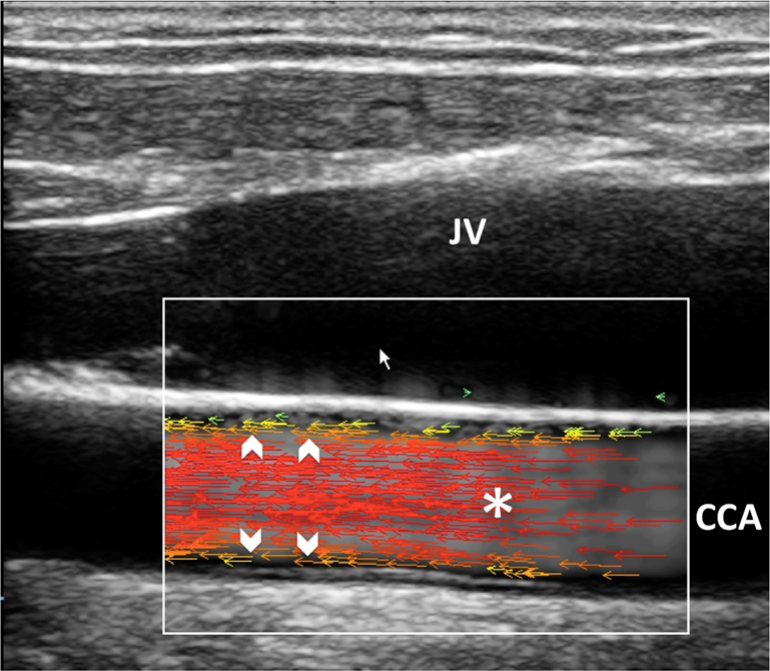
VFI of a straight CCA. Blood flows in thin parallel layers (*red long vectors*; *). The fluid near the boundary (*orange/green vectors*) moves at low velocity (*arrowheads*). CCA = Common carotid artery. JV = Jugular vein

**Fig. 4:**
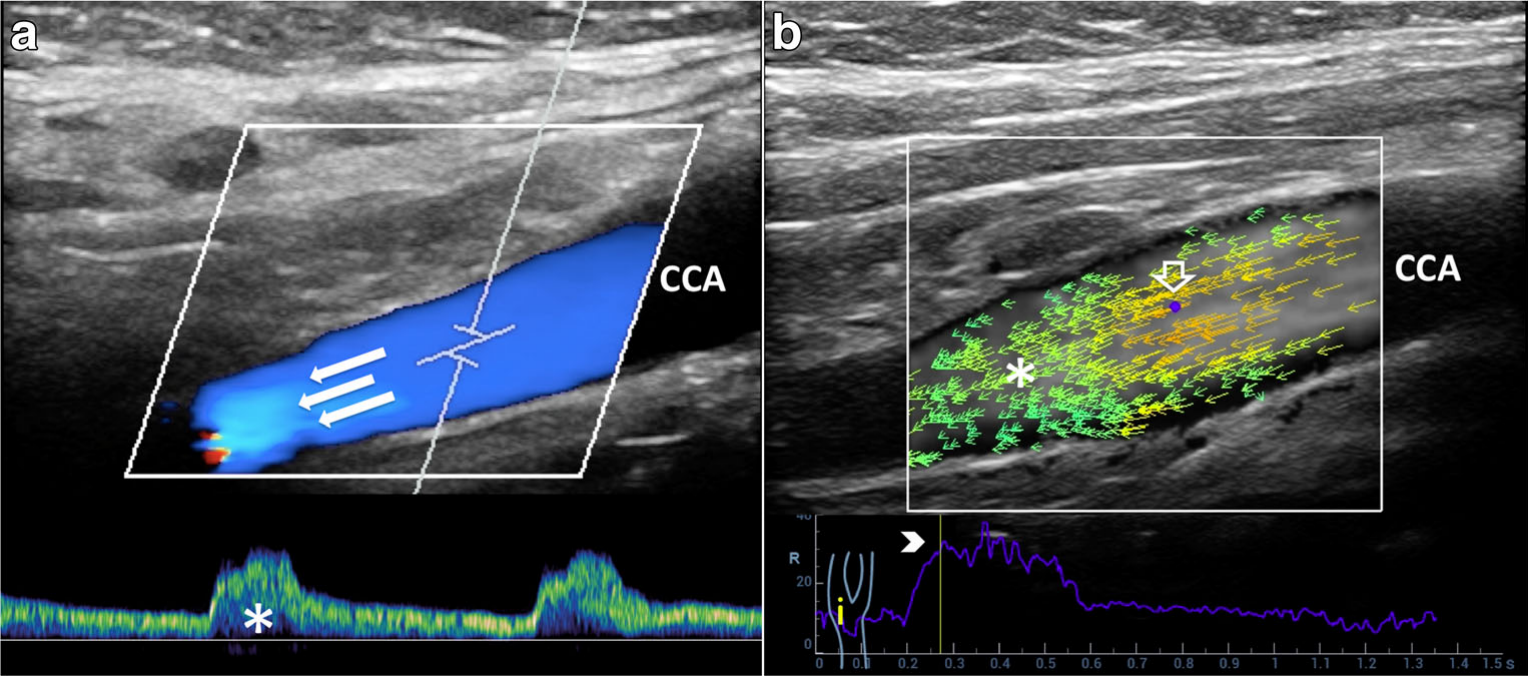
Laminar flow assessment. **a**) CD and PW showing a flow pattern with parabolic profile (*arrows*) and minor spectral broadening (*), respectively. **b**) VFI at the peak systole shows the maximum velocity vectors. Low-velocity vectors (*) of the previous diastole. Maximum velocity point (*void arrow*). Yellow line (*arrowhead*): the moment the VFI frame was acquired. CCA = Common carotid artery

In straight vessels, VFI clearly shows the velocity vectors flowing in parallel layers, with no disruption between the layers (Fig. 3); the layers toward the middle tend to flow faster compared to the boundary layers. Despite the correct representation of the real streamlines’ movements, VFI does not add any new specific information, for diagnostic purpose, compared to the conventional US techniques (Fig. 4).

#### VFI disturbed flow pattern’s relationship with the vessel geometry

A specific feature of the carotid bifurcation is the anatomic sinus at the origin of the internal carotid artery. The anatomical variations of the bifurcation angle and curved vessels can also increase the amount of flow instability. These aspects can lead to the detachment of the layers forming the laminar flow resulting in complex flow. Though flow reversal may be considered a normal phenomenon as suggested by some authors [8], it is well-known that, beyond a certain limit, disturbed flow may increase plaque formation [3, 4]. Thus, a technique such as VFI, able to recognize disturbed flow, may shed new light on the differences between pathological and physiological conditions.

**Fig. 5:**
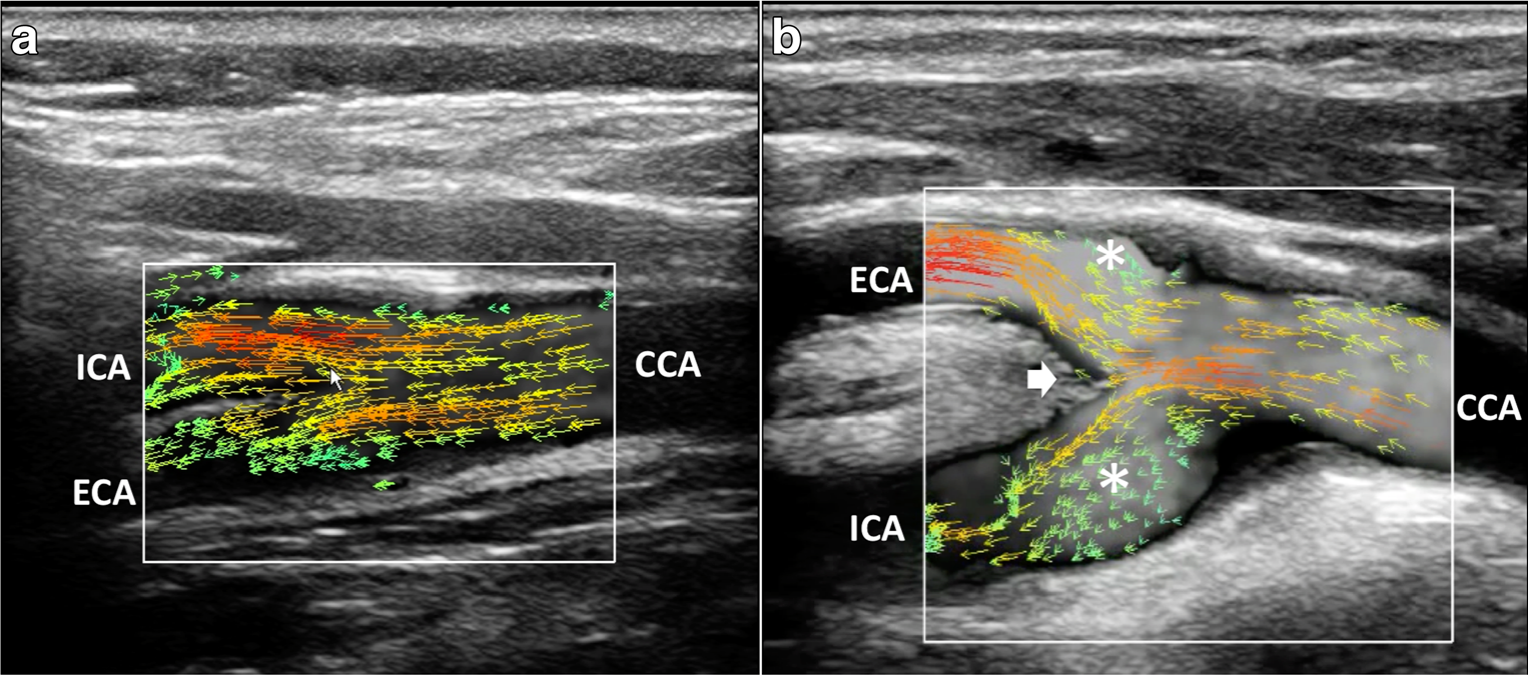
VFI patterns in CB various shapes. **a**) Tight angle between ICA and ECA: streamline laminar flow (*red and orange vectors*) during systole. **b**) Wide angle (> 45°) between ICA and ECA. The maximum velocity red vectors in the bulb hit the flow divider (*short arrow*), leading to the detachment of layers and generating vortex flow (*). CCA = Common carotid artery. ICA = Internal carotid artery. ECA = External carotid artery

**Fig. 6:**
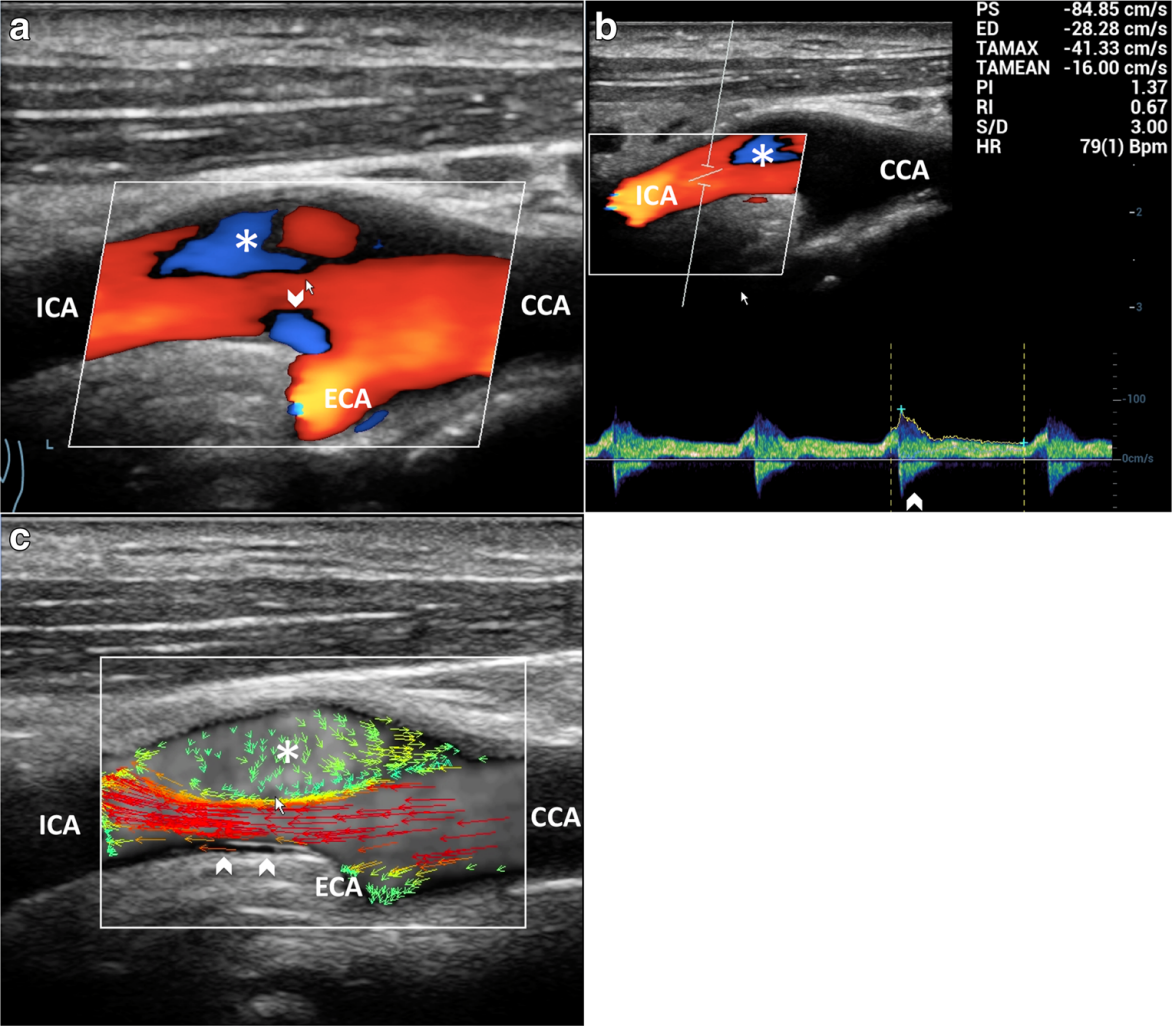
Reverse flow in enlarged ICA sinus. **a**) CD shows reverse flow (*) along the opposite side to the divider and at their apex (*arrowhead*). **b**) PW: bidirectional flow (*arrowhead*), distally to the CD reverse flow (*), suggesting wider extension of vorticity. **c**) VFI outlines a high-velocity red vectors streamline (*arrowheads*) and a wide low flow vorticity (*). CCA = Common carotid artery. ICA = Internal carotid artery. ECA = External carotid artery

**Fig. 7:**
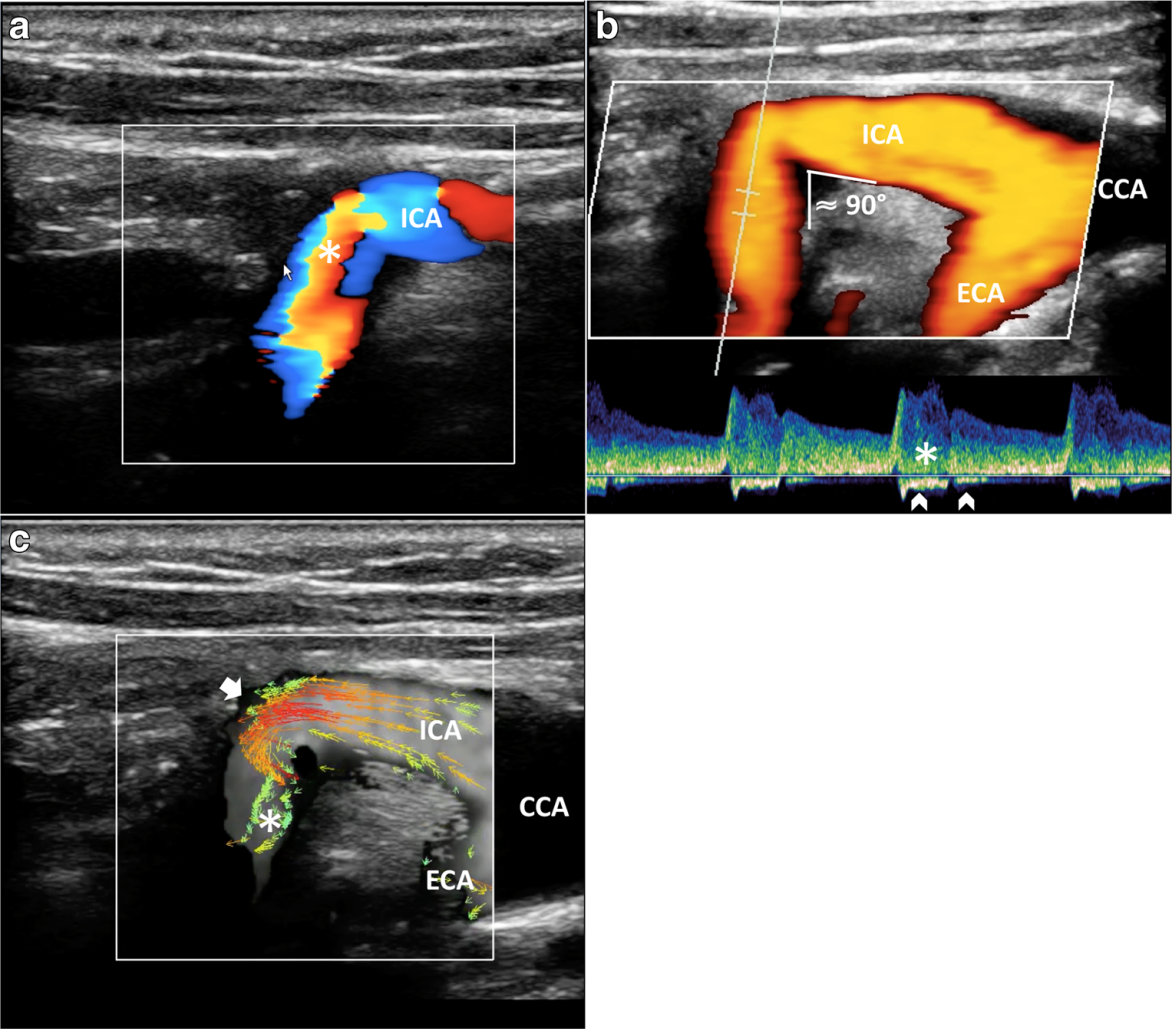
Flow assessment in kinked ICA (angle ≈90°). **a**) CD shows a mosaic of colors—aliasing artifact (*). **b**) Power Doppler outlines the lumen reduction (*arrow*), without any quantification. PW bidirectional flow (*arrowhead*) and spectral broadening (*) highlight the turbulence. **c**) VFI quantifies the stricture by showing high-velocity red vectors (*short arrow*) and strong vorticity (*). CCA = Common carotid artery. ICA = Internal carotid artery. ECA = External carotid artery

Contrary to conventional US methods (Fig. 1), which, at an inclination of 90 degrees, do not provide visualization and quantification of the flow complexity, VFI outlines the streamline’s behavior in different anatomical conditions, such as bifurcation (Fig. 5a, Vid 5a), enlargement of carotid sinus (Fig. 6) and vessel kinking (Fig. 7, Vid 7c). Flow disturbances highlighted by VFI became more evident in case of shape variations of the carotid bifurcation, for example, when the bifurcation angle between the ICA and ECA is larger than 45°. In such a condition, VFI shows the flow streamlines axially aligned in the CCA hitting the flow divider and causing complex flow both in the ICA and the ECA (Fig. 5b, Vid 5b).

#### VFI disturbed flow pattern’s relationship with atherosclerosis

CD cannot quantify the velocity and precise direction of the streamlines, but is very useful in the detection of areas of abnormal blood flow, which are investigated further using the spectral Doppler technique. Nevertheless, in presence of a low-degree stenosis, a condition in which the increase of velocity is not relevant, the role of conventional US Doppler technique is less evident. However, considering that plaque formation and their progression may be a result of disturbed flow, further characterization of the complex flow patterns should be investigated.

**Fig. 8:**
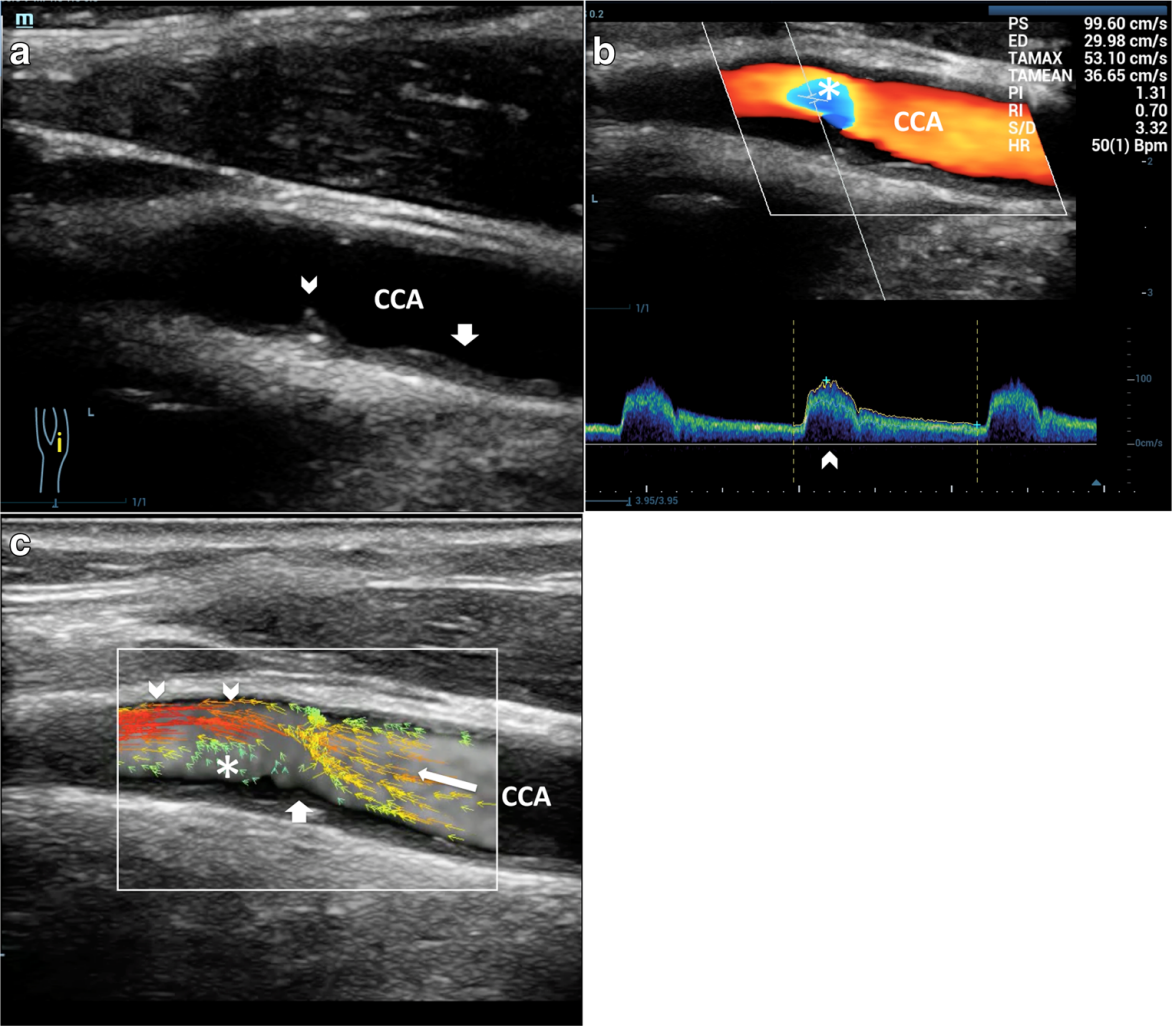
Flow patterns in low-degree stenosis. **a**) B-mode demonstrates atherosclerotic thickness (*short arrows*) and a rose thorn minor plaque (*arrowhead*). **b**) CD shows a limited area of increased velocity (*). PW excludes a relevant PSV (99 cm/s) and spectral broadening (*arrowhead*). **c**) VFI shows aminar flow before the plaque (*arrow*), layers detachment from the boundary (*short arrow*), eccentric max velocity vectors (*arrowhead*) and large complex flow (*) along the opposite wall. CCA = Common carotid artery

**Fig. 9:**
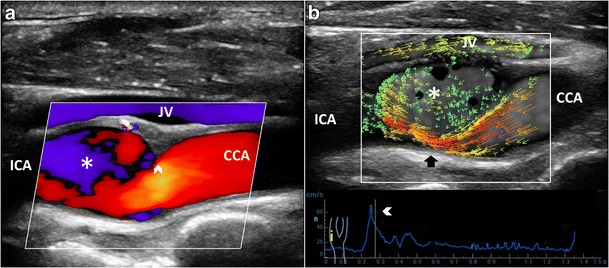
Flow vorticity in low-degree stenosis and vessel dilatation. **a**) B-mode depicts a rose thorn plaque (*arrowhead*) before an enlarged bulb (*); CD shows a mixture of velocities and directions. **b**) VFI quantifies a large vortex (*short green vectors*) (*) during systolic deceleration (*arrowhead*). High-velocity vectors streamline toward the posterior wall (*black arrow*). CCA = Common carotid artery. ICA = Internal carotid artery. JV = Jugular vein

In this condition, VFI allows better understanding of the streamlines progression and vorticity formation and depicts the relevant hemodynamic influence of some plaques, which would otherwise be considered non-significant when using conventional US techniques (Fig. 8, Vid 8c and Fig. 9, Vid 9a, 9b). These findings might help to explain why plaque instability and rupture commonly occur in lesions with less than 50% stenosis, as outlined by Shields [1].

**Fig. 10:**
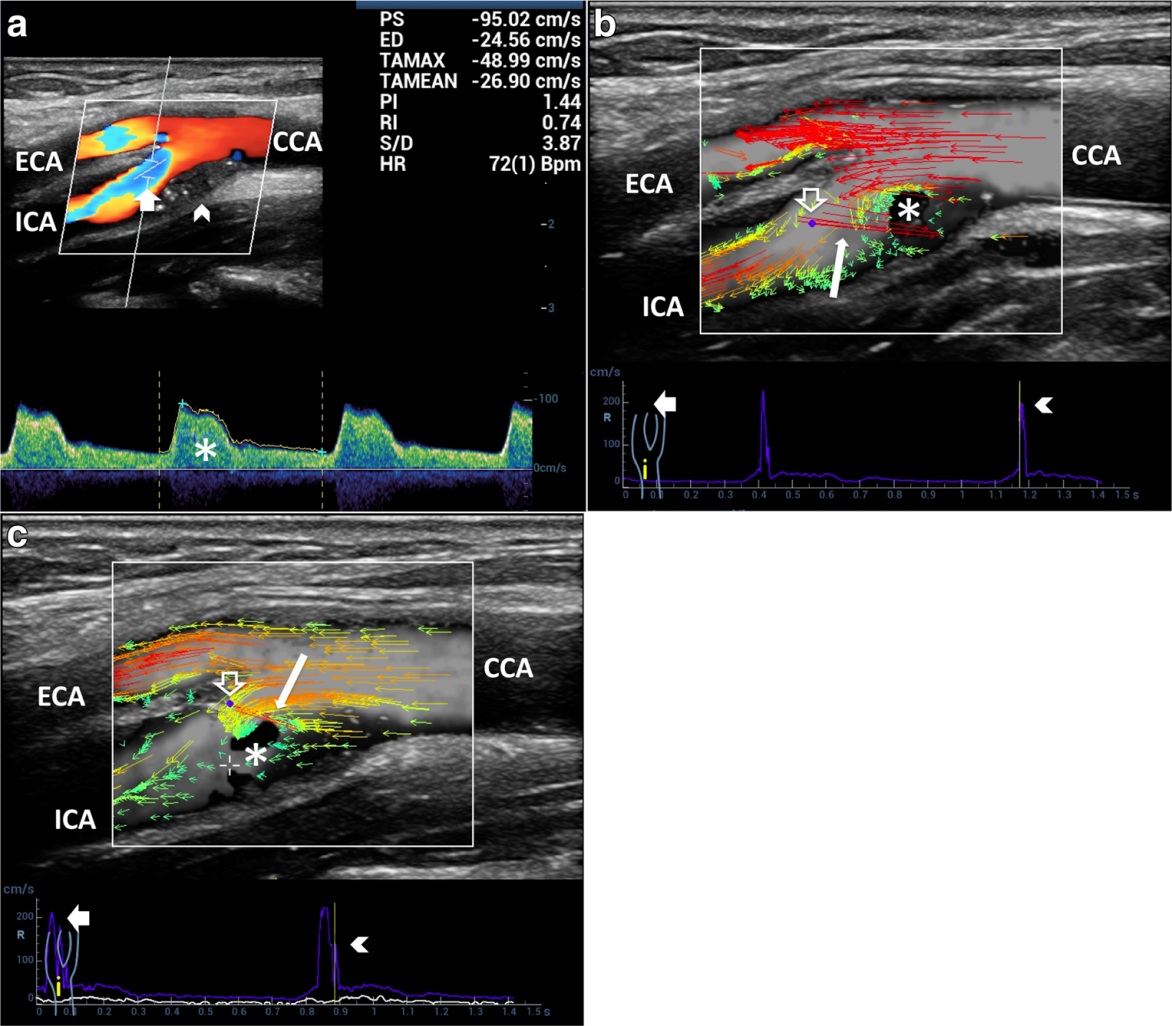
Flow patterns in low-degree to moderate stenosis. **a**) B-mode and CD of the CB show a plaque (*arrowhead*) and increased velocities in the ICA (*short arrow*), respectively. PW measures a moderate velocity and spectral broadening (*). **b)** VFI at low PRF, at the peak systole (*arrowhead*) shows high-velocity red vectors. Long red vectors due to aliasing (*arrow*), start at the blue dot (*void arrow*), move back. Vector velocity (*white arrow*) is higher than the measured PW. **c)** At high PRF, VFI displays the higher velocities at the stenosis level (*orange/yellow vectors*). Reverse red vectors (*arrow*) start from the blue dot (*void arrow*). In **b**), **c**) recirculation depicted by green vectors (*). CCA = Common carotid artery. ICA = Internal carotid artery. ECA = External carotid artery

**Fig. 11:**
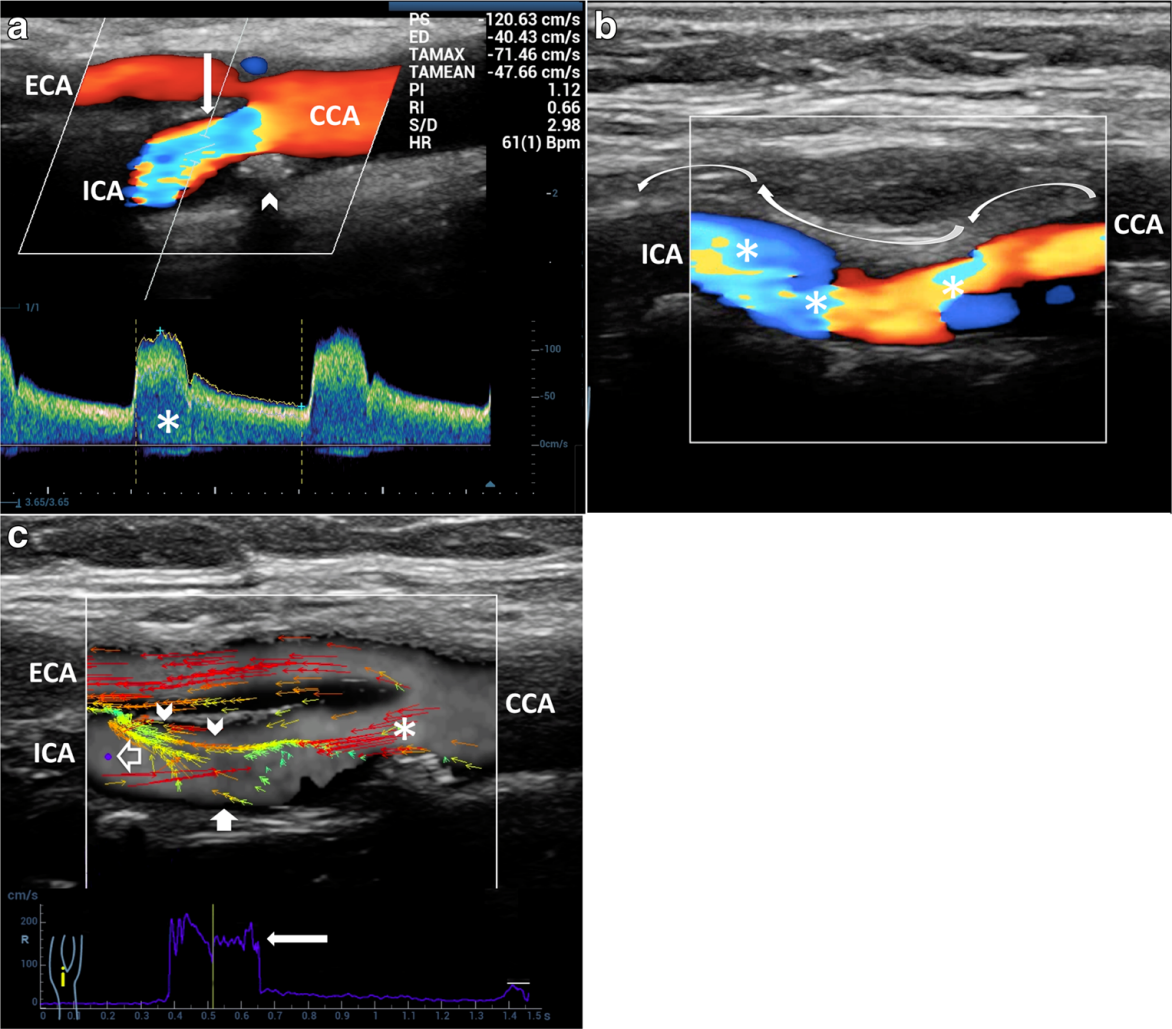
Flow patterns in moderate stenosis. **a**) B-mode and CD of the CB show an eccentric plaque (*arrowhead*; 50%–60% diameter stenosis) and increased velocities (*white arrow*). PW depicts an increased velocity (PSV 120 cm/s) and spectral broadening (*). **b)** Distally to the stenosis (*arrow*), CD shows a mixture of colors (*) due to aliasing and to the non-linear vessel course (*curved arrows*). **c**) VFI during systole: high-velocity vectors (*) at the stenosis; orange/yellow medium velocity vectors (*arrowheads*) and complex flow below: low-velocity green vectors and reverse flow red vectors (*white short arrow*). Turbulence duration (*arrow*). CCA = Common carotid artery. ICA = Internal carotid artery. ECA = External carotid artery

Even in low-degree to moderate stenosis, in which the local increase of velocity can be easily detected by conventional US Doppler techniques, VFI provides a better understanding of disturbed flow extension and duration, useful in the medical management of carotid stenosis (Fig. 10, Vid 10b, 10c). The vorticity assessment becomes more evident in case of moderate stenosis . VFI may detect the randomly and rapidly fluctuating velocities, characteristic of the turbulent flow, varying in subsequent cardiac cycles (Fig. 11, Vid 11b, 11c).

**Fig. 12:**
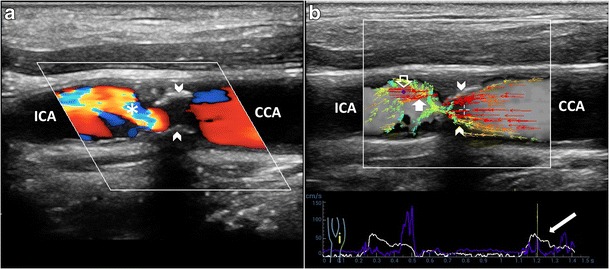
Flow patterns in hemodynamically relevant stenosis. **a**) B-mode and CD of the ICA sinus demonstrates a short plaque (*arrowheads*) causing severe stenosis: CD depicts a mixture of high velocities and directions (*), distally to the stenosis. **b**) VFI shows high-velocity vectors bundled (*arrowheads*) while entering the stenosis. Distally, turbulent flow coded by green/orange vectors, randomly oriented, and by reversed red vectors (*short white arrow*). Blue dot (*void arrow*): max velocity point. CCA = Common carotid artery. ICA = Internal carotid artery

In case of hemodynamically relevant stenosis, the combination of various criteria, such as PSV and EDV or ratio of internal to common carotid PSV, is commonly used to define the critical clinical value. However, sometimes there is a considerable overlap with moderate stenosis. In such condition, the relevant hemodynamic consistency may be highlighted using VFI: the deformation of the streamline flow profile at the entrance of the stenosis, and the amount of turbulence distal to the plaque, can express the seriousness of the critical stenosis (Fig. 12, Vid 12b).

The comparison of technical characteristics between CD and VFI is shown in Table 1.

**Table 1:** Comparison of technical characteristics between color Doppler and high-frame rate VFI

	Color Doppler	High-frame rate VFI
**Parameters**		
Acquisition technique	Line-by-line	Multiangle plane waves
Scan time	Real time	1.5 s
Temporal resolution	20–24 fps	400–600 fps
Flow visualization frame rate	20–24 Hz	20–30 Hz
Qualitative evaluation	+	+
Spatial resolution	+	++
Flow direction estimation	Monodirectional	Multidirectional
Velocity estimation	Mean velocity	True velocity magnitude
Velocity vector measurement	Axial component	Axial & lateral components
Quantitative evaluation	−	+
Beams angle dependence	Dependent	Independent
Hemodynamics	+	++
Aliasing artifact	+	+

### VFI limitations

As any method based on pulse repetition frequencies, even VFI, suffers from aliasing, which limits the possibility to study high-grade stenosis. Velocity scale on the system must be adapted to the hemodynamic findings in order to limit the artifact (Fig. 10, Vid 10b, 10c).

At the moment, VFI, as a two-dimensional (2D) technique, does not allow a precise comprehension of the actual three-dimensional (3D) shape of the streamline. A 3D vector flow method, which allows very high temporal resolution, will be needed to completely elucidate the complexity of hemodynamic patterns.

Despite the quantitative information on the flow, related to the vector velocities calculation, the evaluation of complex flow with VFI is still visual, thus subjected to intra- and inter-observer variability. To overcome this limitation, some quantitative tools will be necessary. Vortex extension and duration must be taken into account; even vector concentration as suggested by Pedersen et al. should be considered [29].

## Conclusions

Conventional US methods have the ability to measure blood velocities and flow direction on the basis of the Doppler principle. Doppler US is angle-dependent, only estimates the axial component of blood flow velocity and is limited by the vessel geometry. This issue represents a significant limitation when applied to a “non straight” vessel such as the carotid bifurcation, the site of a vorticity pattern in the majority of subjects. VFI, as a quantitative method assessing disturbed flow patterns of the carotid bifurcation, has the potential to allow better understanding of the diagnostic value of complex flow and to enhance risk stratification.

## Electronic supplementary material

Vid 2a (AVI 4634 kb)

Vid 2c (AVI 17943 kb)

Vid 5a (AVI 13907 kb)

Vid 5b (AVI 19533 kb)

Vid 7c (AVI 18471 kb)

Vid 8c (AVI 15156 kb)

Vid 9a (AVI 5549 kb)

Vid 9b (AVI 15125 kb)

Vid 10b (AVI 14632 kb)

Vid 10c (AVI 14403 kb)

Vid 11b (AVI 16507 kb)

Vid 11c (AVI 18507 kb)

Vid 12b (AVI 17671 kb)
